# Incidence of Typhoid and Paratyphoid Fevers Among Adolescents and Adults in Yangon, Myanmar

**DOI:** 10.1093/cid/ciy1109

**Published:** 2019-03-07

**Authors:** Win Thandar Oo, Tin Ohn Myat, Wah Win Htike, James E Ussher, David R Murdoch, Kay Thi Lwin, Min Zaw Oo, Michael J Maze, Hla Hla Win, John A Crump

**Affiliations:** 1 Department of Microbiology, University of Medicine 1, Yangon, Myanmar; 2 Centre for International Health, University of Otago, Dunedin, New Zealand; 3 Southern Community Laboratories, Dunedin Hospital; 4 Department of Immunology and Microbiology, University of Otago, Dunedin; 5 Department of Pathology, University of Otago, Christchurch, New Zealand; 6 Department of Preventive and Social Medicine; 7 Department of Medicine, University of Medicine 1, Yangon, Myanmar; 8 Department of Medicine, University of Otago, Christchurch, New Zealand

**Keywords:** incidence studies, Myanmar, paratyphoid fever, typhoid fever, typhoid vaccine

## Abstract

**Background:**

Accurate estimates of typhoid disease burden are needed to guide policy decisions, including on vaccine use. Data on the incidence of enteric fever in Myanmar are scarce. We estimated typhoid and paratyphoid fever incidence among adolescents and adults in Yangon, Myanmar, by combining sentinel hospital surveillance with a healthcare utilization survey.

**Methods:**

We conducted a population-based household health care utilization survey in the Yangon Region 12 March through 5 April 2018. Multipliers derived from this survey were then applied to hospital-based surveillance of *Salmonella* Typhi and Paratyphi A bloodstream infections from 5 October 2015 through 4 October 2016 at Yangon General Hospital (YGH) to estimate the incidence of typhoid and paratyphoid fevers among person ≥12 years of age.

**Results:**

A total of 336 households representing 1598 persons were enrolled in the health care utilization survey, and multipliers were derived based on responses to questions about healthcare seeking in the event of febrile illness. Of 671 Yangon residents enrolled over a 1-year period at YGH, we identified 33 (4.9%) with *Salmonella* Typhi and 9 (1.3%) with *Salmonella* Paratyphi A bloodstream infection. After applying multipliers, we estimated that the annual incidence of typhoid was 391 per 100 000 persons and paratyphoid was 107 per 100 000 persons.

**Conclusions:**

Enteric fever incidence is high in Yangon, Myanmar, warranting increased attention on prevention and control, including consideration of typhoid conjugate vaccine use as well as nonvaccine control measures. Research on incidence among infants and children, as well as sources and modes of transmission is needed.

*Salmonella enterica* serovars Typhi and Paratyphi A are the major causes of enteric fever. *Salmonella* Typhi and Paratyphi A are human host-restricted pathogens transmitted by fecally contaminated water and food, estimated to cause 11.8 and 3.8 million illness [1] and 128 200 and 25 200 deaths [2], respectively, worldwide in 2016. These global estimates, although underpinned by an increasing number of studies from diverse locations, still lack robust data on disease incidence, complications, and deaths from many countries. Although both typhoid and paratyphoid fevers remain common in many countries South and Southeast Asia [3–7], declining incidence have been reported from some countries [8, 9] although data have been limited from others [10].

In October 2017, the World Health Organization (WHO) Strategic Advisory Group of Experts on immunization recommended typhoid conjugate vaccine for routine use in children over 6 months of age in typhoid endemic countries, and in December 2017 the first typhoid conjugate vaccine was prequalified by WHO [11]. Improved data on typhoid fever epidemiology are needed to inform country-level decisions about vaccine adoption and strategies. In Myanmar, outbreaks of typhoid fever have been reported regularly since 1989 [12, 13]. However, there have been few reports of hospital-based studies of community-acquired bloodstream infections [10]. Studies among outpatients and inpatients in Mandalay 2012–13 [14] and among inpatients in Yangon 2015–16 [15] have demonstrated that *Salmonella* Typhi and Paratyphi A are leading causes of bacteremia. However, to our knowledge, no studies have estimated the incidence of enteric fever due to *Salmonella enterica* serovars Typhi and Paratyphi A. Combining sentinel site surveillance with healthcare utilization surveys is an established method of estimating disease incidence in resource-limited settings [16, 17].

In order to provide data on typhoid fever incidence in Myanmar, we conducted a healthcare utilization survey (HCUS) in the Yangon General Hospital (YGH) catchment area and sentinel surveillance for enteric fever at YGH.

## METHODS

### Study Location

**Figure 1.**
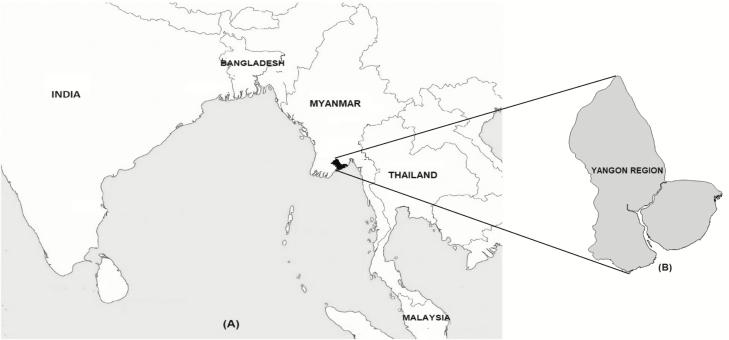
Map of South and Southeast Asia showing (*A*) Myanmar and (*B*) Yangon Region.

With a population of 7 360 703, the Yangon Region is the most populous in Myanmar (Figure 1). YGH is a 2000-bed public civilian tertiary referral hospital for adolescents and adults serving Yangon Region.

### Healthcare Utilization Survey

#### Household Selection

In the first stage, 48 wards were selected at random proportional to population size from the 689 wards of Yangon Region based on the 2014 Myanmar Population and Housing Census [18]. In the second stage, 336 households were selected by simple random sampling from ward household lists obtained from each selected Township Health Department.

#### Design and Administration of the Survey

The healthcare utilization survey was conducted from 12 March through 5 April 2018. After obtaining informed consent, members of the study team administered the survey to the heads of 7 households in each ward. The standardized survey was adopted from other widely used healthcare utilization questionnaires [19, 20] and included questions about demographics, socioeconomic status, and healthcare seeking behavior. Healthcare seeking questions asked separately about usual healthcare seeking behavior in the event of fever <3 days and ≥3 days duration by age groups <5 years, 5–<12 years, and ≥12 years, as well as actual healthcare seeking behavior of any individual household members experiencing fever in the past 3 months. Choices included YGH and other public and private hospitals and health centers in Yangon, as well as drug stores, traditional healers, self-treatment, and nothing.

### Surveillance for Community-acquired Bloodstream Infections

As part of a study of the etiology of febrile illness in Yangon, patients aged ≥12 years admitted during weekdays to medical units of YGH were prospectively enrolled from 5 October 2015 through 4 October 2016. Methods and results have been previously described [15]. In brief, patients admitted to the adult medicine wards were eligible for enrolment if they had an oral temperature of ≥38.0ºC on admission and had been admitted for <24 hours. Demographic information, including the participant’s township and ward of residence, were collected. Following cleansing of the skin with denatured ethanol and povidone iodine, venous blood was drawn, and 8–10 mL was inoculated into standard aerobic blood culture bottle (bioMérieux, Marcy l’Etoile, France) and sent to the Clinical Microbiology Laboratory, YGH, for incubation, isolation, identification, and antimicrobial susceptibility testing (AST). Identification and AST of isolates was confirmed at Southern Community Laboratories, Dunedin, New Zealand.

#### Laboratory Methods

Blood culture bottles were assessed for volume adequacy by comparing the weight before and after inoculation. BacT/ALERT standard aerobic bottles were loaded into the BacT/ALERT 3D Microbial Detection system (bioMérieux, Marcy l’Etoile, France) and incubated for 5 days. Isolation, identification, and AST of organisms were done by VITEK 2 compact 60 system (bioMérieux, Marcy l’Etoile, France). Identification and AST was confirmed by matrix-assisted laser desorption/ionization time-of-flight mass spectrometry (MALDI-ToF-MS) (Microflex LT, Bruker Daltonics, Billerica, Massachusetts, USA) and the Phoenix Automated Microbiology System (Becton and Dickenson, Franklin Lakes, NJ, USA) at Southern Community Laboratories, Dunedin, New Zealand. To confirm *Salmonella* serovars, whole genome sequencing was performed at New Zealand Genomics Ltd, and data were analyzed at the Department of Microbiology and Immunology, University of Otago, Dunedin, New Zealand using the Nullarbor pipeline [21].

### Incidence Calculation

**Figure 2.**
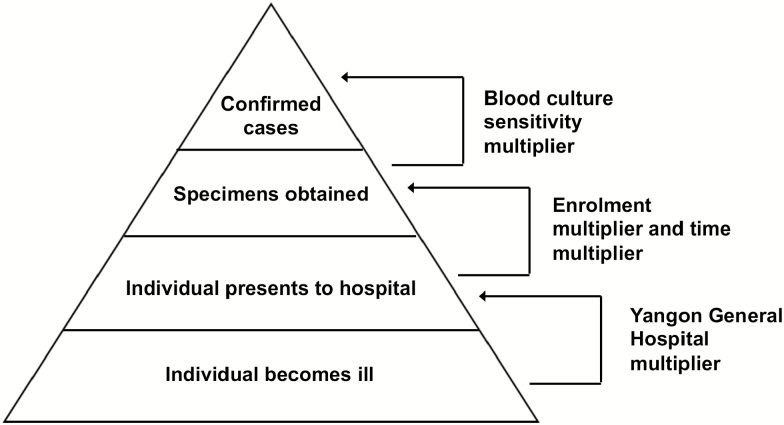
Surveillance pyramid showing multipliers used to account for incomplete case identification. Modified from Crump et al [16].

We estimated incidence with the use of multipliers derived from the healthcare utilization survey and the community-acquired bloodstream infection study. Multipliers accounted for persons with typhoid and paratyphoid fever who would potentially be missed through the stages of reporting including healthcare facility choice, referral from another inpatients facility, and diagnostic test sensitivity. Multipliers are the multiplicative inverse of the relevant proportions (Figure 2). We calculated the “YGH multiplier” to account for healthcare seeking preferences and cases potentially missed due to selection of healthcare providers or options not under surveillance. The YGH multiplier was derived based on responses from heads of households to HCUS questions: “Where would household members usually seek healthcare if they had fever ≥3 days duration?” We selected the first and second choice responses to “fever for ≥3 days” as most representative of where patients sufficiently ill to warrant hospital admission would seek care. We validated responses to questions about usual healthcare seeking questions against actual healthcare seeking of household member who had fever ≥3 days in the past 3 months.

#### Derivation of Multipliers

We calculated “time multiplier” to account for enrolment occurring on 5 of 7 days per week. In addition, we calculated the enrollment multiplier to account for patients who were eligible but not enrolled in fever surveillance for any reason. We also calculated a sensitivity multiplier to reflect the sensitivity of single blood culture for diagnosis of typhoid and paratyphoid fever when compared to bone marrow aspirate culture [22, 23]. We calculated the referral multiplier to account for the patients transferred to YGH from other inpatients facility assuming that transfer may not reflect a patient’s preference of healthcare facility. Calculation of the YGH multiplier, enrollment multiplier, sensitivity multiplier, and referral multiplier are shown in results.

### Sensitivity Analysis

We performed 1-way sensitivity analysis, repeating our estimates of incidence for varying proportions of the population that would visit YGH with febrile illness.

### Statistical Analysis

Data were stored and analyzed using STATA, version 15.1 (STATA-Corp, College Station, TX, USA). Incidence calculations and sensitivity analysis were carried out using Microsoft Excel 2016 (Microsoft Corporation. Redmond, WA, USA). One-way sensitivity analysis was performed using the upper and lower bounds of the 95% confidence interval of the hospital multiplier derived from a binomial exact test for the question “Where would household members usually seek healthcare when a household member had fever for ≥3 days?” Validation of “usually healthcare seeking” against “actual healthcare seeking” was calculated with comparison of proportion test using STATA, version 15.1.

### Research Ethics

This study was approved by the Research and Ethics Review Committee of University of Medicine 1 and Department of Medical Research, Myanmar, and the University of Otago Human Ethics Committee, New Zealand.

## RESULTS

### Healthcare Utilization Survey

We enrolled 336 households, including 1598 household members. Among selected households, 57 (16.9%) refused to participate and were replaced with another randomly selected household. All households had at least 1 member ≥20 years of age, 148 (44.0%) households had at least 1 member aged from 12 through 19 years of age, and 144 (42.9%) households had at least 1 member aged <12 years of age. Of those interviewed, 1 (0.3%) of 336 head of the households chose YGH as the healthcare facility that household members ≥12 years of age usually seek healthcare if they had fever ≥3 days duration and formed the basis of the YGH multiplier.

Of 1598 household members responding, 237 (14.8%) reported having fever in the past 3 months. Of those reporting fever, 1 (0.4%) person sought care from YGH. The difference in proportion of “usually healthcare seeking” against “actual healthcare seeking” behavior of the community using YGH as healthcare facility in case of fever was not statistically significant (*P* = .84).

#### YGH Multiplier Derivation

The proportion of household heads responding that adolescent and adult household members usually seek healthcare for fever ≥3 days duration at YGH, and the resultant multipliers are shown in Table 1.

**Table 1.** Multipliers Based on Responses to Relevant Questions in Healthcare Utilization Survey, Yangon Region, Myanmar, 2018

Age, Years	Households	YGH	YGH Proportion	YGH Multiplier
Where would household members usually seek healthcare if they had fever of ≥3 days?				
≥ 12	336	1	0.0030	336
≥ 12–19	148	1	0.0068	148
≥ 20	336	1	0.0030	336

Abbreviation: YGH, Yangon General Hospital.

### Fever Surveillance

A total of 947 patients consented and were enrolled in the community-acquired bloodstream infection surveillance study [15]. Among 947 participants, 671 (70.9%) resided in the Yangon Region, 170 (17.8%) were adolescents aged 12–19 years, and 777 (82.2%) were adults aged >19 years. Of 671 patients from Yangon Region, *Salmonella* Typhi was isolated from the blood cultures of 33 (4.9%) patients and *Salmonella* Paratyphi A from 9 (1.3%). Of 947 blood culture bottles collected, 850 (89.8%) had adequate blood volume.

#### Multiplier Derivation

Of patients screened for fever surveillance, 1045 were eligible for enrollment, and 947 (90.6%) were enrolled. Among those enrolled, 170 (90.4%) of 188 eligible adolescents and 777 (90.7%) of 857 eligible adult patients were enrolled in the study, resulting in an overall enrollment multiplier of 1.1. We also calculated a sensitivity multiplier of 2.0 to reflect the sensitivity of single blood culture for diagnosis of typhoid and paratyphoid fever, estimated as 50% when compared to bone marrow aspirate culture. Of febrile patients from Yangon Region admitted at YGH, 22 (19%) of 115 adolescents and 86 (15%) of 556 adults reported transfer from other inpatient hospitals of the Yangon Region. Therefore, we adjusted crude case numbers for adolescents and adults at YGH by multiplying with referral multiplier of 0.81 and 0.85, respectively.

### Incidence Calculations

We estimated the 2015–16 annual incidence of enteric fever among adolescents and adults in the Yangon Region as 498 per 100 000 population, with typhoid incidence in this age group 391 per 100 000 persons and paratyphoid incidence 107 per 100 000 persons. Typhoid and paratyphoid incidence among adolescents was 360 per 100 000 persons and 65 per 100 000 persons, respectively, whereas typhoid and paratyphoid incidence among adults was estimated 395 per 100 000 persons and 114 per 100 000 persons, respectively. Further details of incidence calculations and results are shown in Table 2.

**Table 2.** Enteric Fever Incidence Estimates, Yangon Region, Myanmar 2015–2016

	Age, Years	YGH Confirmed Cases	Sensitivity Multiplier	YGH Multiplier	Time Multiplier	Enrollment Multiplier	Referral Multiplier	Annual Cases	Population	Case per 100 000 Persons
Enteric fever	≥12	42	2	336	1.4	1.1	0.84	36 626	7 360 703	498
	≥12–19	13	2	148	1.4	1.1	0.81	4815	1 131 867	425
	≥20	29	2	336	1.4	1.1	0.85	25 591	4 911 502	521
Typhoid fever	≥12	33	2	336	1.4	1.1	0.84	4074	7 360 703	391
	≥12–19	11	2	148	1.4	1.1	0.81	19 414	1 131 867	360
	≥20	22	2	336	1.4	1.1	0.85	28 778	4 911 502	395
	≥12	9	2	336	1.4	1.1	0.84	7848	7 360 703	107
Paratyphoid fever	≥12–19	2	2	148	1.4	1.1	0.81	741	1 131 867	65
	≥20	7	2	336	1.4	1.1	0.85	5597	4 911 502	114

Abbreviation: YGH, Yangon General Hospital.

### Sensitivity Analysis

The results of the 1-way sensitivity analysis are presented in Table 3. We estimated that the annual incidence of typhoid fever ranged from 72 to 14 480 cases per 100 000 population and the estimated annual incidence of paratyphoid varied from 20 to 3949 cases per 100 000 population.

**Table 3.** Sensitivity Analysis of Enteric Fever Incidence Estimate, Yangon Region, 2015–2016

		Age, Years	Households	YGH % (Lower, Upper 95% CI) With Binomial Exact	YGH Multipliers (Lower, Upper 95% CI)	Case per 100 000 Persons
Where household members would usually seek healthcare if they had fever of ≥3 days?	Enteric fever	≥12	336	0.3 (0.008, 1.6)	336 (12 444, 62)	92–18 429
		≥12- 19	148	0.7 (0.02,3)	148 (493, 33)	95–1417
		≥20	336	0.3 (0.008,1.6)	336 (12 444, 62)	96–19 297
	Typhoid fever	≥12	336	0.3 (0.008, 1.6)	336 (12 444, 62)	72–14 480
		≥12- 19	148	0.7 (0.02, 3)	148 (493, 33)	80–1199
		≥20	336	0.3 (0.008, 1.6)	336 (12 444, 62)	73–14 639
	Paratyphoid fever	≥12	336	0.3 (0.008, 1.6)	336 (12 444, 62)	20–3949
		≥12- 19	148	0.7 (0.02, 3)	148 (493, 33)	15–218
		≥20	336	0.3 (0.008, 1.6)	336 (12 444, 62)	21–4221

Abbreviations: CI, confidence interval; YGH, Yangon General Hospital.

## DISCUSSION

We estimate that enteric fever incidence among adolescents and adults in Yangon, Myanmar, exceeds 100 per 100 000 persons per year, the widely accepted threshold for “high” enteric fever incidence [8, 24]. Our findings place enteric fever incidence in Yangon in a similar range to that observed in other high incidence cities in South and Southeast Asia. The annual incidence of typhoid fever in Kolkata, India, and Karachi, Pakistan has been estimated at 214 and 452 per 100 000 population, respectively, in 2004 [8]. Naheed et al reported that the incidence of blood culture-confirmed typhoid fever in Dhaka, Bangladesh in 2003, was 200 per 100 000 person-years [5].

Although our study was restricted to those of adolescents and adult age groups, the incidence of typhoid fever high incidence population is usually highest among infants and young children [24]. Therefore, it is conceivable that typhoid and paratyphoid fever incidence may be considerably higher among infants and children in Yangon than the 391 and 107 per 100 000 persons per year, respectively, estimated among adolescents and adults. Further research to estimate the incidence of typhoid fever among infants and children in Yangon is needed to inform the best strategy for typhoid conjugate vaccine use. Typhoid conjugate vaccine introduction to the routine child immunization schedule might be warranted if the incidence observed in older age groups is matched or exceeded among infants and young children.

To our knowledge, the major sources and modes of transmission of typhoid and paratyphoid fever in Yangon are unknown. However, data from other major cities in South and Southeast Asia highlight the major role that waterborne transmission plays in urban areas with aging municipal water and sanitation facilities [25–27]. Yangon’s water supply is from 4 large reservoirs as well as tube wells, lakes, and ponds. Parts of Yangon’s water reticulation system dates to the early 1900s. Yangon’s water treatment plants use aeration, flocculation, sedimentation, and sand filtration. However, treated water quality is low, and aging reticulation systems may allow inflow of fecally contaminated environmental material during periods of inadequate water pressure [28, 29]. Street-vended foods may also be important vehicles for typhoidal *Salmonella* transmission [26, 27] and is subject to less regulatory control compared to stationary food stalls [30].

Although our findings represent our best effort to estimate typhoid and paratyphoid incidence data for Yangon Region, we recognize a number of limitations. We chose multiplier methods to estimate incidence as limited resources prohibited active surveillance in the entire population. Although the multiplier method is a widely accepted approach to incidence estimation [16, 20], our estimates are based on a relatively small number of cases, and some variation due to random error may occur. In addition, multiplier methods rely on many assumptions. In particular, we assumed that those presenting to YGH with fever were representative of those presenting to other hospitals and healthcare facilities in Yangon Region. Furthermore, our healthcare utilization survey showed that YGH was an uncommon first or second choice to seek healthcare for prolonged fever in Yangon. The small proportion of community members using YGH for fever increases the uncertainty of our incidence estimate, something that we explored and expressed in 1-way sensitivity analysis. Sites for fever surveillance are best chosen in light of the healthcare utilization survey results, rather than *a priori* as was the case in our study [16]. We also assumed that there was no difference between patients who were enrolled and those who were eligible and not enrolled. Finally, the lack of surveillance at healthcare facilities providing pediatric care meant that we were unable to estimate incidence in the critical infant and child age groups.

In conclusion, typhoid and paratyphoid fever incidence in Yangon is high among adolescents and adults and may reflect even higher unmeasured incidence among infants and children. Typhoid conjugate vaccines present a new opportunity to provide long-lasting protection against typhoid fever from infancy and early childhood [31, 32] and should be considered in Myanmar. However, further research to understand the epidemiology of typhoid fever among infants and children is warranted. Of concern, existing vaccines would not prevent paratyphoid fever which is also a major problem in Yangon. This finding highlights the need for development of polyvalent *Salmonella* vaccines [9, 31, 33]. Epidemiologic research to understand the major sources and modes of transmission of enteric fever in Yangon is needed to inform nonvaccine control measures. We would suggest that such research should investigate the role of sanitation facilities, drinking water, and street-vended food. Our findings will help with planning of enteric fever control in Myanmar, including considerations for vaccine introduction.

## Notes

***Acknowledgments.*** The authors thank those involved in recruitment, laboratory work, data management, and study administration, including: Daw Sawthu Nander who leads the healthcare utilization survey in Yangon Region as well as healthcare utilization survey team members Ye Min Kyaw and others. They thank the YGH febrile illness study team, including Dr Khine Mar Htun, Dr Khin Khin Kyourk, Dr Su Hnin Aung, Dr Su Htet Aung, Dr Su Myat Aye, Dr Sai Nay Linn Htet, Dr Si Thu Sein Win, Dr Htet Htet Lin, Dr Htet Htet Lwin, Dr Win Thit Lwin, Dr Lin Thit Lwin, and Dr Hein Htet Aung for collecting data and blood samples, and Dr Hla Kyae Mone and Dr Khaing Mar Oo for assisting laboratory evaluations. They thank Dr Khwar Nyo Zin, Consultant Microbiologist, Clinical Microbiology Laboratory, YGH for use of laboratory facilities and sharing her experience, and Dr Chuen Yen Hong, University of Otago, to undertaking independent randomization for the study. They also thank Professor Paul N. Newton and Associate Professor Mayfong Mayxay from Lao-Oxford-Mahosot-Hospital-Wellcome Trust Research Unit, Microbiology Laboratory, Mahosot Hospital, Vientiane, Lao PDR for assisting with study design and questionnaires development for healthcare utilisation survey. They thank the study participants as well as the clinical staff and administration at YGH for their support during this study.

***Financial support.*** This research was supported by New Zealand Health Research Council through the e-ASIA Joint Research Program (grant 16/697) and University of Otago Development Grant. W. T. O. and T. O. M. received support through University of Otago Doctoral Scholarships. M. J. M. received support from the Frances G. Cotter scholarship through the University of Otago.

***Supplement sponsorship.*** This supplement is sponsored by the Center for Vaccine Development and Global Health (CVD) at the University of Maryland School of Medicine.

***Potential conflicts of interest*. **All authors: No reported conflicts. All authors have submitted the ICMJE Form for Disclosure of Potential Conflicts of Interest. Conflicts that the editors consider relevant to the content of the manuscript have been disclosed.
